# Supplementary material for: Structural basis of nucleosome transcription mediated by Chd1 and FACT
Source: Nat Struct Mol Biol. 2021 Apr 12;28(4):382–7. doi: 10.1038/s41594-021-00578-6 (PMC8046669; doi:10.1038/s41594-021-00578-6)
Supplement: Supplementary file 1 — Sequence of nucleosomal construct. [file 41594_2021_578_MOESM1_ESM.pdf]

nature portfolio

https://doi.org/10.1038/s41594-021-00578-6

Supplementary information

Structural basis of nucleosome transcription mediated by Chd1 and FACT

In the format provided by the authors and unedited

Final PCR product:

Non-template DNA
Template DNA

TspRI cut site

ACGAAGCGTAGCATCACTGTCTTGTGTTTGGTGTGTCTGGGTGGTGGCCGTTTTCGTTGTTTTTTTCTGTCTCGAACCTGGAGACTAGGGAGTAATCCCCTTGGCGGTTAAAACGCGGGGGACAGCGCGTACGTGCGTTTAAGCGGTGCTAGAGCTGTCTACGACCAATTGAGCGGCCTCGGCACCGGGATTCTGAT

TGCTTCGCATCGTAGTGACAGAACACAAACCACACAGACCCACCACCGGCAAAAGCAACAAAAAAAGACAGAGCTTGGACCTCTGATCCCTCATTAGGGGAACCGCCAATTTTGCGCCCCCTGTCGCGCATGCACGCAAATTCGCCACGATCTCGACAGATGCTGGTTAACTCGCCGGAGCCGTGGCCCTAAGACTA
