# Supplementary figures and images for: Structural basis of nucleosome transcription mediated by Chd1 and FACT
Source: Nat Struct Mol Biol. 2021 Apr 12;28(4):382–7. doi: 10.1038/s41594-021-00578-6 (PMC8046669; doi:10.1038/s41594-021-00578-6)

**Fig. 1b**

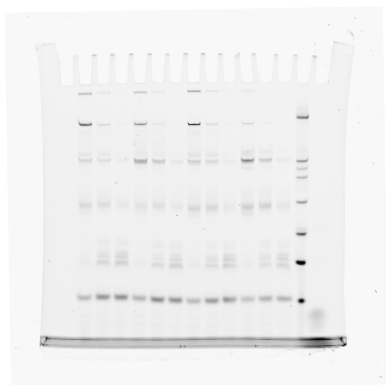

Replicate 1

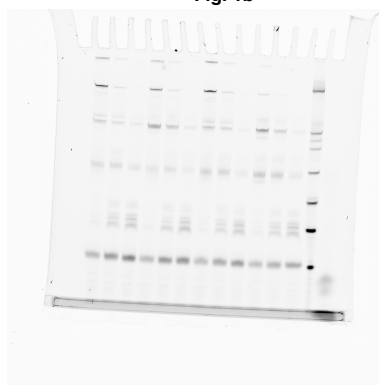

Replicate 2

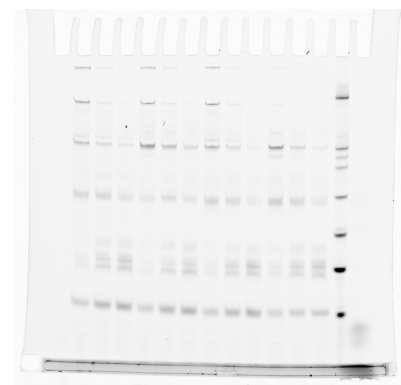

Replicate 3

Supplement: Source Data Fig. 1 — Source gels of denaturing gels. [file 41594_2021_578_MOESM6_ESM.pdf]

Extended Data Fig. 2b

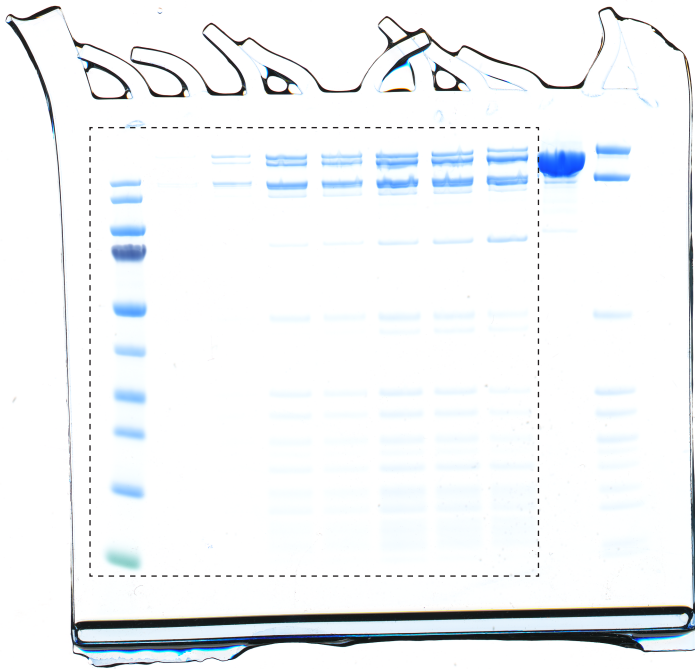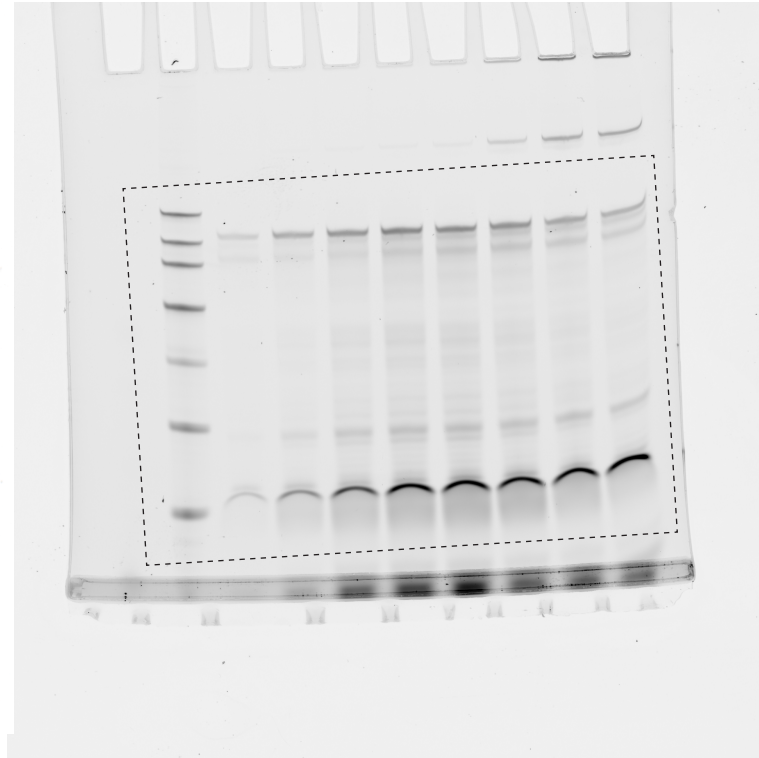

Extended Data Fig. 2e

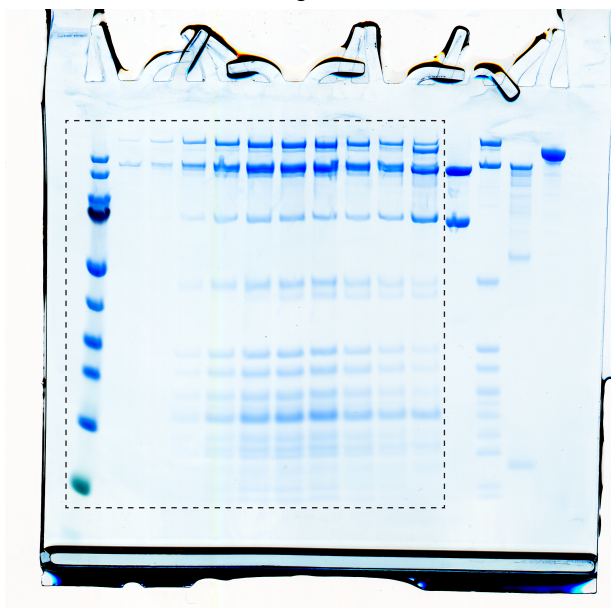

Extended Data Fig. 2f

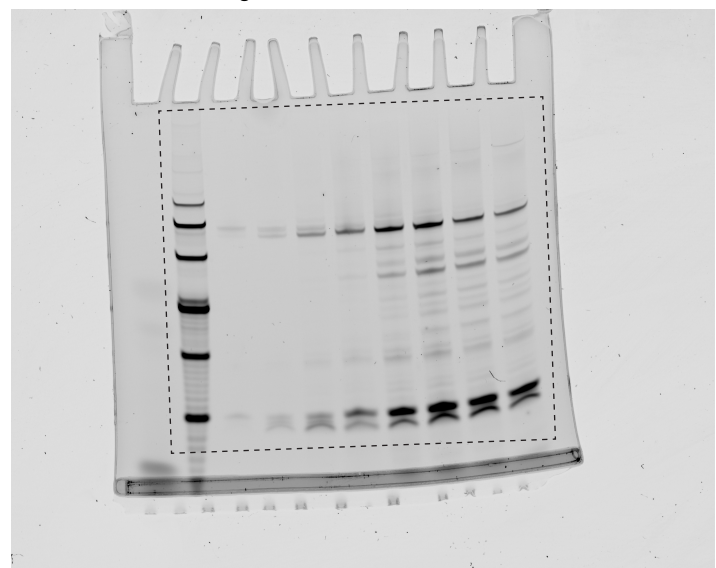

Supplement: Source Data Extended Data Fig. 2 — Source gels of SDS−PAGE and denaturing gels. [file 41594_2021_578_MOESM8_ESM.pdf]

Extended Data Fig. 9

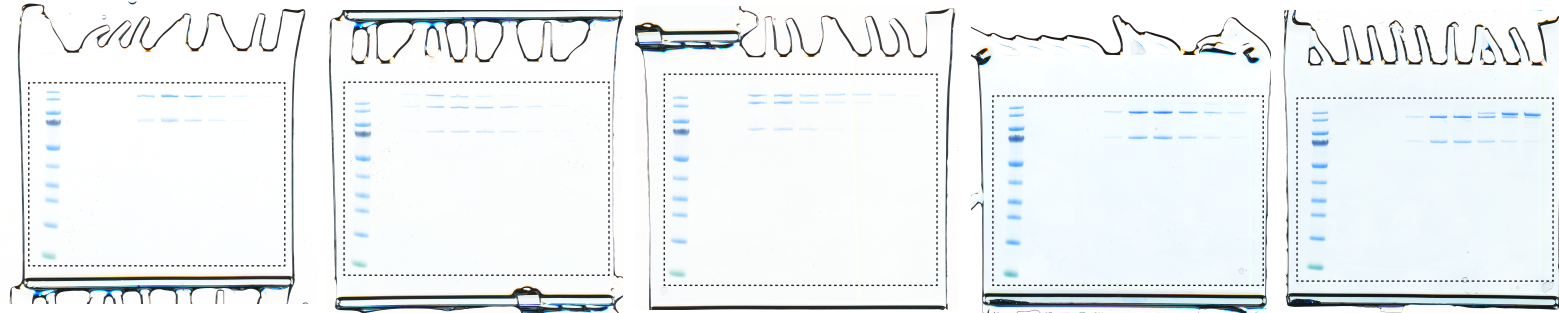

Supplement: Source Data Extended Data Fig. 9 — Source gels of SDS−PAGE gels. [file 41594_2021_578_MOESM9_ESM.pdf]
